# Negative psychological aspects of working with experimental animals in scientific research

**DOI:** 10.7717/peerj.11035

**Published:** 2021-04-20

**Authors:** Hanna Mamzer, Agnieszka Zok, Piotr Białas, Mirosław Andrusiewicz

**Affiliations:** 1 Faculty of Sociology, Adam Mickiewicz University in Poznań, Poznań, Greaterpoland, Poland; 2 Division of Philosophy of Medicine and Bioethics, Poznan University of Medical Sciences, Poznań, Greaterpoland, Poland; 3 Chair and Department of Cell Biology, Poznan University of Medical Sciences, Poznań, Greaterpoland, Polska

**Keywords:** Negative psychological aspects, Scientists, Animals, Laboratories, Experiments, Handling

## Abstract

The aim of the study was to reveal the negative psychological aspects of using animals by scientists and to determine whether the emotional tensions and stress are associated with performing experiments on animals. All 150 participants of the study conduct experiments on animals in their work. Computer-assisted web interviewing, was used to collect the data. Correlation matrices for factorial analysis of main component loads and cluster analysis have been calculated as grouping methods revealed two different categories of researchers, which were mostly distinguished by acceptance and aversion to animal testing and animal welfare. The main findings demonstrated, that there is a group of respondents who feel discomfort when performing experiments on animals. Especially young people involved in animal testing, feel remorse, emotional tension and helplessness.

## Introduction

The progressive changes, in relation to humans and other animals (Rollin, 2002; Rollin, 2005; Rollin, 1989; Wise, 2006; Wise, 2005), observed worldwide since the 1970s of the twentieth century include diverse areas such as: industrial livestock production and sourcing of the livestock products (Singer, 1975), promotion of plant-based diets, attitudes towards companion animals (Katcher & Beck, 1983) and farm animals (Le Neindre et al., 2009), use of animals for entertainment but also conducting experiments on animals (Singer, 2001). The latter issue is also gaining importance in the scientific circles (Greek, 2004), where an increasing polarisation of approaches can be observed: from those that radically reject the use of animals for experiments to those that assume that animal testing cannot be discontinued. This disagreement is accompanied by an increasing concern about the possibility of introducing and implementing the ethical standards of conducting experiments on animals. This issue is particularly important given the growing interest of widely understood public opinion and the media in the methods of implementing the experiments (Festing & Wilkinson, 2007; Matthiessen, Lucaroni & Sachez, 2003; Market and Opinion Research International, 2002; Market and Opinion Research International, 2005).

Public interest in animal experimentation stems from different motivations (Clemence & Leaman, 2016): the desire to monitor how public funds are disbursed, the desire to monitor the well-being of animals used in experiments, the desire to understand what scientific research consists of and its implications for the society. All this results in a situation where animal experimentation is becoming an important social issue with increasing interest among non-professionals (Hobson-West, 2010). The self-awareness of members of the civil society therefore increases the willingness to scrutinise the activities of scientists and researchers.

Changes in human-animal relations also alter the attitudes of society towards the way in which scientific experiments on animals should be conducted and whether they should be carried out at all—not because of their merit, but because of the fact that animals suffer and are killed during experiments (Publications Office of the EU, 2016; Market and Opinion Research International, 1999). This raises more and more objections and comments, although it should be recognised that the average citizen has little knowledge of what is occurring in the scientific laboratories (Hobson-West, 2010).

Changes in the approach of people towards animals are also observed in the attitudes of people that conduct experiments on animals and deal with their deaths and the implementation of procedures. The changes in relation to the animals which are subject of experiments are very strong—if one compares today’s perception of “who an animal is” with the perception of animals proposed by Descartes, a tremendous chasm becomes apparent. Over the course of three centuries, this perception has changed from equating animals with machines to perceiving them as humans (compare The Nonhuman Rights Project) (Gross & Tolba, 2015). An in-depth reflection on animal experimentation is therefore important for different communities but also for society as a whole. Within the so-called “one health” concept, which indicates that the health of humans and other animals is closely related, the worlds of humans and non-humans are intertwined and it is not just a humanistic rhetorical figure. It is demonstrated by phenomena linked to the appearance of zoonoses such as African swine fever, avian influenza, bovine spongiform encephalopathy and recently SARS-CoV-2 (Destoumieux-Garzón et al., 2018; Tang, Comish & Kang, 2020).

Although the reflection on the methods and general sense of animal experimentation is needed primarily to ensure animal welfare, another important reason for in-depth reflection on this subject is the welfare of the people conducting the experiments. We were wondering if the experiments have a negative psychological impact, because they involve inflicting pain on animals and dealing with their death. The results presented below are unprecedented as currently there are no scientific researches demonstrating the consequences of animal experimentation for the experimenters themselves. However, research is being carried out on veterinarians, who have direct contact with the suffering and death and the killing of animals. This research indicates that the veterinary profession is prone to significant negative psychological effects as a result of the exposure to animal suffering resulting even in suicide (Rollin, 2000; Stoewen, 2015; Platt et al., 2010). Our research was aimed at answering a research issue formulated as the following question: “Does testing on animals have negative psychological effects on the experimenters?”.

## Materials & methods

### Participants and procedures

One hundred and fifty participants were recruited on the Internet in Poland from June 2019 until August 2019. The selection of respondents was purposeful: a hyperlink to the survey was sent via two institutions related to conducting animal experiments: The National Ethical Committee and the PolLASA (Polish Laboratory Animal Science Association). The survey was anonymous, and voluntary responses were given. The survey was conducted in Polish. The computer-assisted web interview presented information about the study background information and questions regarding personal feelings about animal testing procedures. To participate in the study, only the criterion of working with animals had to be fulfilled. Within the study group, subgroups of respondents were distinguished regarding gender and the second division was based on factor analysis of relation of questionnaire answers.

### Statistical Analyses

Statistical analysis was performed using Statistica version 13 for Windows OS (TIBCO Software, Tulusa, OK, USA). Results from the 150 surveys were summarized using descriptive statistics. Correlation matrix for factor analysis of principal components loadings for multi-select multiple-choice questions (see Supplemental Materials) unrotated factor rotation was computed to obtain homogenous subgroups. It was computed for nine variables with unrotated factor rotation. Missing data were casewise deleted, 150 cases were processed and 105 valid cases were accepted. Due to the requirements of statistical software, incomplete questionnaires were excluded from statistical analysis. Based on the multi-select multiple-choice questions, using the analysis of Ward’s method cluster joining and Euclidean distances, it was possible to extract two clusters for the questionnaire part regarding accompanying feelings (procedures performed on animals during studies). Missing data were casewise deleted. The obtained clusters were then used for further grouping. The distributions of continuous variables were assessed with the Shapiro–Wilk test. For the studies, the median and quartile distributions were determined for the values in the rank scale. These measures were compared between the subgroups Mann–Whitney *U* test. The distribution of nominal data were compared between different groups and subgroups described earlier with Chi-square tests accordingly Cochran’s Rule. The correlation coefficient (R) was determined by Spearman’s rank correlation tests. According to Gellman and Hill (A. Gellman and J. Hill, Data Analysis Using Regression and Multilevel/Hierarchical Models, Cambridge University Press, New York, NY, USA, 2007.), analysis adjustment for multiple analyses is not necessary for an exploratory model building context. Thus, the researchers used a *P* < 0,05 level of significance for all analyses.

### Survey limitations and strengths

The presented survey has some limitations. First, the survey covered only national statistics (only the Polish population) to limited extend—only for peoples and institutions who had contact with the National Ethical Committee and the PolLASA. Because the e-mail addresses of many researchers are in both databases, we cannot precisely determine the number of people who received the message. Taking under consideration the typical CAWI survey, average survey response rates could be estimated as high as 30% for online forms. Second, the survey did not have formal validation by expert and public pilot testing because this was the first research on this topic. During research it also appeared that it is impossible to provide statistical data on actual number of people conducting experiments on animals. We believe that this is a minor concern for this survey aimed at a very specific set of animal-use issues. The response return without missing data suggests that the questions were understood by people participating in the survey.

This study has several strengths. It is the first research of this type in Poland. The participants were selected purposefully, and was designed to obtain responses from representative researchers who execute procedures on animals. The study aimed to reveal the psychological aspects of conducting experiments on animals for people experimenting and to examine whether the emotional tensions and stress accompany actual conducting of experiments on animals. This proves that not only animals suffer in experiments, but also humans are subject to negative influence of conducted procedures on their psychological welfare.

## Results

Most of the respondents were women. However, the percentage of sex did not differ statistically (*P* > 0.05). There was no statistically significant difference between men and women in age, time of experimentation and invasiveness of procedures as well as the number of animals used in procedures (*P* > 0.05). However, in the case of cluster analysis, the respondents differed only in the invasiveness of procedures (*P* = 0.005). Among the people qualified for cluster 1, invasiveness was declared as slighter (Table 1). The participants experimented with differ groups of animals (agnathan, fishes, amphibians, reptiles (22%), small-size mammals—e.g. rodents (56%), medium-sized mammals—e.g., rabbit (7%) and big mammals—e.g., sheep, cows, horses (15%)).

**Table 1:** Descriptive statistics of the surveyed group.

	All participants	Females	Males	Cluster 1*	Cluster 2*
N	150	95 (63%)	55 (37%)	57 (54%)	48 (46%)
Median age (interval)	41–50	31–40	41–50	31–40	41–50
How many years ago were the experiments performed (Me (Q1–Q3))	15 (8–26)	14 (7–22)	16 (10–29)	18 (9–32)	14 (8–21)
Invasiveness (Me (Q1–Q3))	7 (4–9)	7 (4–9)	7 (4–9)	6 (4–8)	8 (5–10)
Number of animals (Me (Q1–Q3))	15 (3–32)	10 (3–30)	15 (3–50)	12 (2–30)	10 (2–40)

**Notes:**

* a—clusters for the analysis of multiselect multiple-choice questions.

N, number of participants; Me, median; Q1–Q3, quartiles.

**Figure 1:**
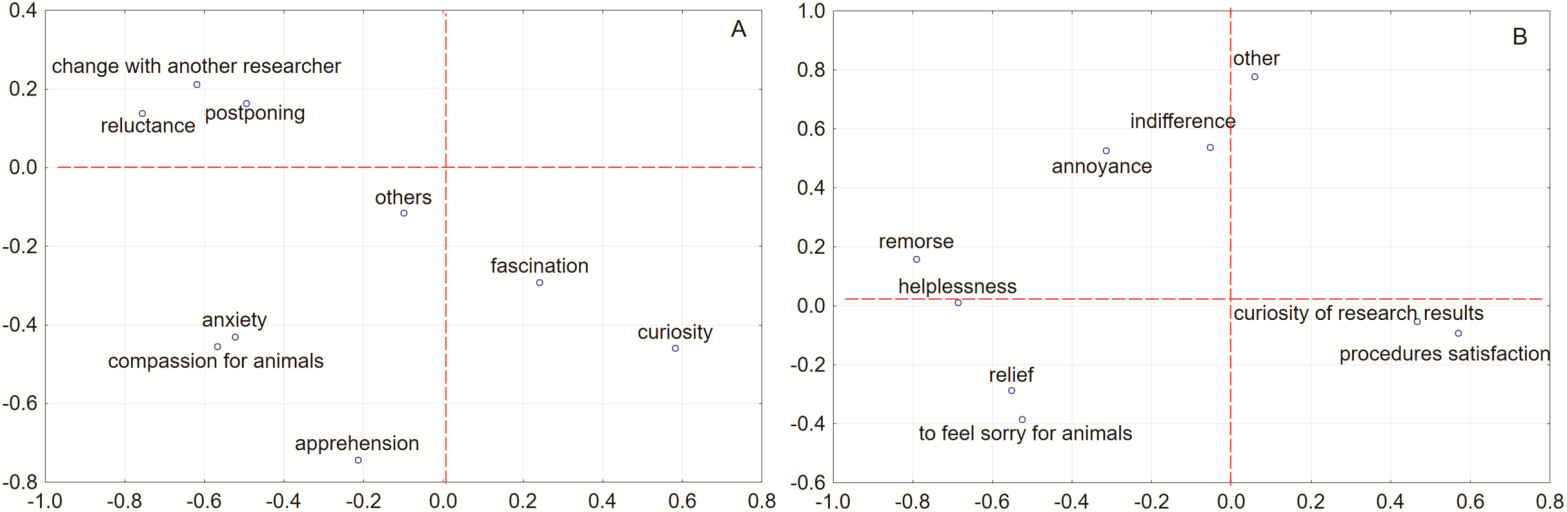
Plot of principal components factor loadings 2D. The correlation matrix for factor analysis of principal components loadings for multi-select multiple-choice questions computed for ten variables with unrotated factor rotation. Analyzing the feelings that appeared in respondents during studies (A) and after the completed research protocol (B).

The correlation matrix for factor analysis showed that the least frequently common answers were reluctance and apprehension (the largest distance between those two answers). The co-occurrence of responses was observed in three groups for the question regarding feelings experienced during procedures performed on animals (at the study time). These groups focused (1) change with another researcher, postponing the exercise in time and reluctance to do it, (2) fascination and curiosity, and (3) anxiety and compassion for animals (the most commonly occurring together) as well as apprehension and other feelings (Fig. 1A). Feelings with a similar profile are grouped together. Negatively correlated feelings are positioned on opposite sides of the plot origin (opposed quadrants). The distance between points and the origin measures the quality of the points on the factor map. Feeling points that are away from the origin are well represented on the factor map.

Analyzing the feelings that appeared in respondents after the completed research protocol, factor components loadings analysis revealed the occurrence of four groups. The least often feelings accompanying the respondents were sorry feeling for animals and answered described as other. The groups received joint answers (1) annoyance, indifference, helplessness, and remorse, (2) relief and sorry feeling for animals, (3) curiosity of research results with simultaneous satisfaction with procedures, and (4) the last group, independent of the others, were other answers given (Fig. 1B).

**Figure 2:**
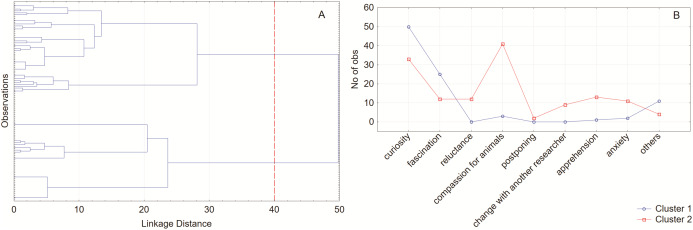
Hierarchical cluster membership tree plot for 105 cases. Joining (tree clustering) Ward`s method and Euclidean distances (linkage distance) (A). Number of observations in each cluster (B). The analysis reveal two clusters differed in the accompanying feelings during studies.

Based on the multi-select and multiple-choice questions, it was possible to extract two clusters used for further grouping. When performing procedures on animals during studies and the accompanying feelings, clusters (almost equal in the case number «54% in cluster 1 and 46% in cluster 2; *P* = 0.57») differed to the greatest extent by compassion for animals (*P* < 0.0001). In cluster 2, more people showing compassion for animals. However, the other answers given in the survey varied between the clusters (Figs. 2A and 2B).

### χ^2^ agglomerations based on animal procedures during studies and accompanying feelings

There was a significant difference in the frequency of the respondents’ sex, qualified for clusters 1 and 2. In cluster 1, the number of women and men was very similar (49% *vs*. 51%), whereas, in cluster 2, the percentage of women was significantly higher (77%). The difference was significant (*P* = 0.003).

Respondents assigned to specific clusters differed in the matter of seeing the possibility of abandoning animal experiments. In both clusters, people mostly did not declare the possibility of resignation from animal experiments (84%). But taking into consideration the percentage ratio in cluster 1 and 2, 5% and 19% respectively declare no possibility of animal procedures resignation (*P* = 0.028).

Considering the inability to avoid animal experiments, 96% of respondents from cluster 1 believe that animal experiments are inevitable, while in cluster 2 this percentage was smaller and amounted to 83% (*P* = 0.049).

When the respondents were asked whether animal experiments are necessary, the responses were not equal in each cluster. In the first cluster, only 33% of respondents believe that animal experiments are unnecessary, while in the cluster 2 as much as 61% of respondents have the same opinion (p=0.01).

Considering the question of whether animal experiments should be carried out at present, Cluster 1 includes people who think that animal experiments should not be conducted, while in Cluster 2, 84% of respondents had similar views (*P* = 0.003).

Respondents between clusters also differed regarding emotional burden when conducting animal experiments. In cluster 1, there were 57% of people indicating emotional load, while in cluster 2 as much as 89% showed a similar answer (*P* = 0.0004).

In cluster 1, the majority of respondents (69%) did not indicate a feeling of remorse due to killing animals, while among respondents from the second cluster 69% pointed to the occurrence of remorse (*P* = 0.0002).

When analyzing the avoidance of procedures performed on animals, in cluster 1 the vast majority (81%) did not avoid such tasks, while in cluster 2 more than half of the respondents (59%) avoided such procedures (*P* < 0.0001).

In both clusters, the respondents were not guided by curiosity in conducting experiments, but the difference was significant in numbers of answers. It was 88% in cluster 1 and 69% in cluster 2 (*P* = 0.017). Most respondents were also not fascinated by experimentation. In cluster 1, the fascination was characterized by 44% in the second 25%. These clusters, however, slightly differed (*P* = 0.044).

The respondents also did not show reluctance to perform animal experiments. In cluster 1, all responded that they did not feel reluctant, while in cluster 2, it was 75% (*P* = 0.00006). On the one hand, the percentage of non-reluctance feeling people was high, but on the other hand, they do perceive their work as unnecessary and stressful.

The respondents in both clusters differed significantly in their feelings of compassion for animals. In cluster 1, up to 96% were not guided by compassion, while in cluster 2, by contrast, 85% felt compassion (*P* < 0.00001).

Respondents could indicate if they avoided experimenting by exchanging with someone else. In cluster 1, none of the respondents declared such a change, while in the second cluster 19% declared such a change (*P* = 0.00056).

There was also a difference in apprehension between those surveyed qualified for the appropriate clusters. In cluster 1, only 2% of respondents showed a sense of concern in cluster 2, it was 27% (*P* = 0.0002).

Similar to fear, a sense of anxiety was distributed. In cluster 1, anxiety was declared by 4% of respondents in cluster 2 it was 23% (*P* = 0.0028).

**Figure 3:**
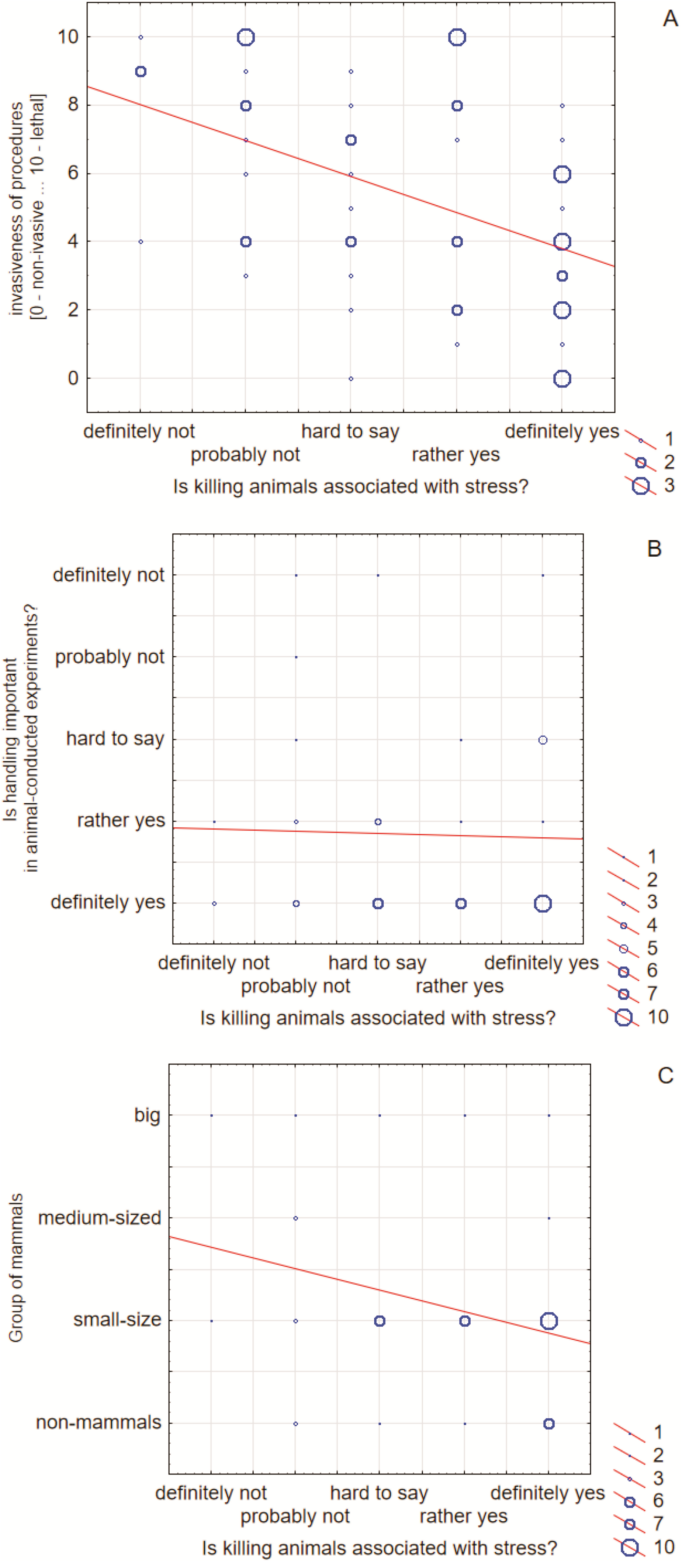
Scatterplot of the stress feeling in respondents. The stress was correlated with invasiveness of the procedures (A), the importance of handling (B) and the group of animals (C).

Spearman rank correlation test revealed an average, directly proportional, statistically significant correlation between the invasiveness of procedures and the stress feeling in respondents (R = 0.37, *P* = 0.005). All data obtained in the study were published in the attached excel file as raw data. Due to the lack of statistical significance, cluster 2 was not described in details in the manuscript body regarding figures and descriptive statistics. The respondents in cluster 1 also saw the relationship between the importance of handling and stress. Along with recognizing the positive importance of handling, the level of stress associated with killing animals increased (R = −0.45, *P* = 0.0008). As before, in the whole group and the cluster 2, there was no significant correlation showed (Fig. 3B). A correlation in cluster 1 was also noted for the group of animals used in the procedures (R = 0.28, *P* = 0.046). The stress feeling decreased if the experiments were carried out by respondents on larger animals and increased rather for lower animals. In the whole group and the cluster 2, there was no significant correlation showed (Fig. 3C).

**Figure 4:**
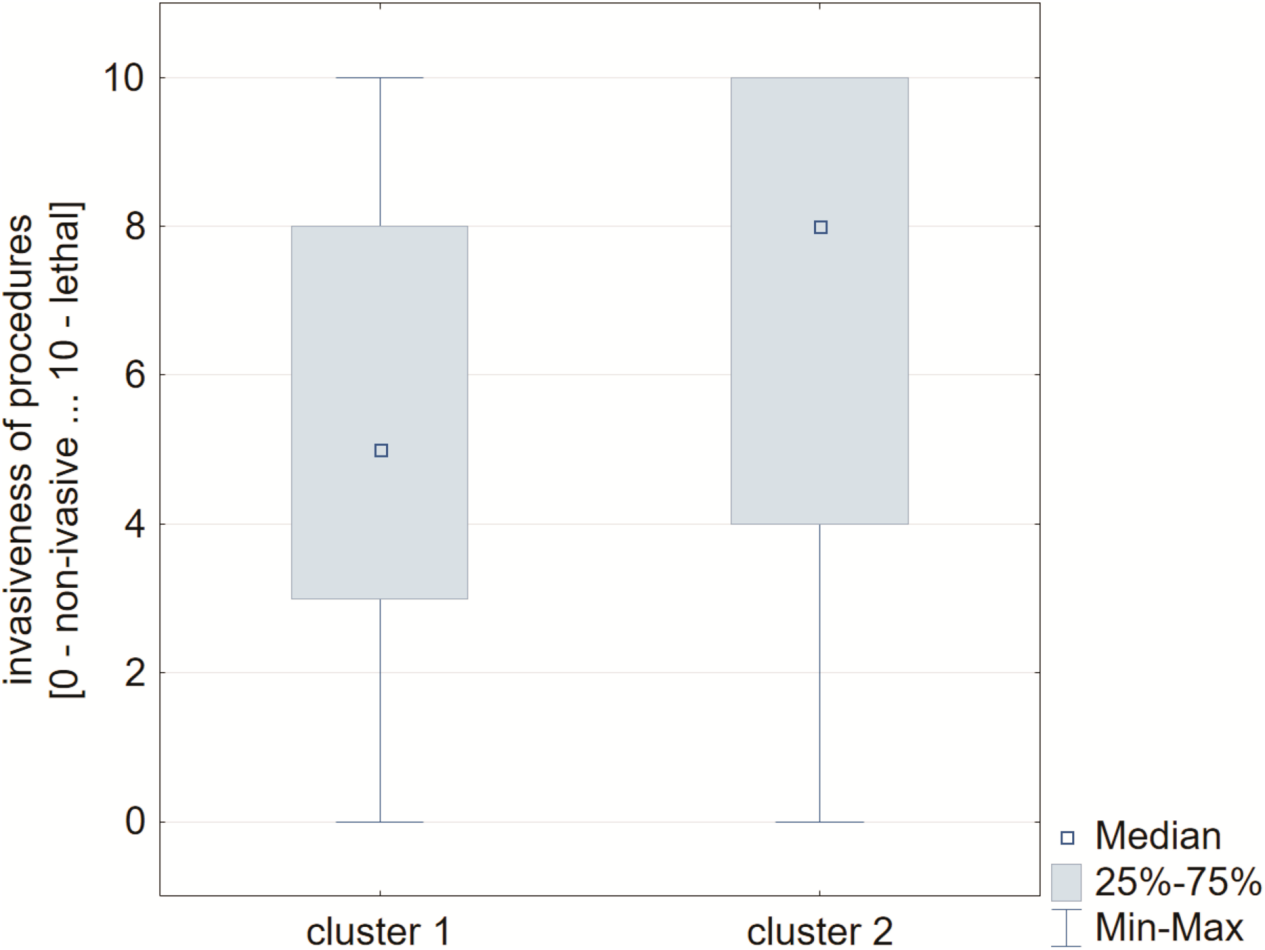
Box-whiskers plot for invasiveness of procedures carried out in distinguished clusters. In the 1^st^ cluster the invasiveness of the procedures was estimated significantly at lower level comparing to those respondents assigned to the 2^nd^ cluster.

Considering the previously described clusters for the analysis of multi-select and multiple-choice questions and performing animal procedures during the study, a statistically significant difference was observed between the invasiveness of the procedures. In cluster 1, the respondents pointed to less invasive procedures compared to cluster 2 (Me = 4.5 [2.5–8] *vs*. Me = 8 [4–10], *P* = 0.004; Fig. 4).

**Figure 5:**
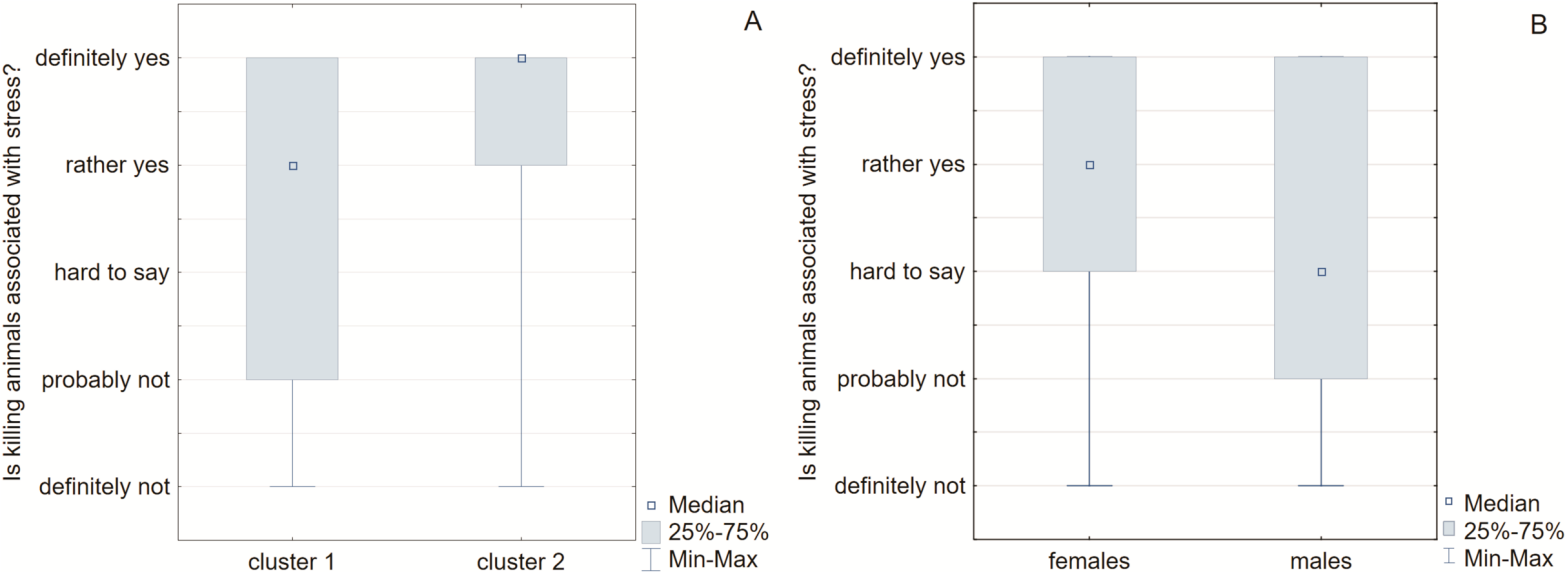
Box-whiskers plot for stress feeling. The groups were distinguished in cluster (A) and gender (B) dependent manner. The females as well as survey participants in 2^nd^ cluster felt intensified stress.

In addition, respondents determining the level of stress associated with killing animals. In cluster 1, they showed a lower level of stress compared to the second cluster, where the respondents felt a significantly higher stress level (*P* = 0.003) (Fig. 5A). Interestingly, the same difference was observed in the case of women and men respondents. The women’s group pointed higher stress feeling than the men (killing animals “rather yes” *vs*. “hard to say”, respectively) (Fig. 5B).

A detailed analysis of the questionnaire was shown in Table 2. Analyzing the respondents’ answers, it was found that men rarely see the possibility of resignation from animal experiments compared to women. 14% of women surveyed considered that there was a possibility of abandoning animal experiments; in the case of men, it was only 4% (*P* = 0.045).

**Table 2:** Questionnaire analysis.

Question	Answer	Female (*N*)	Male (*N*)	*P-*value	Cluster 1 (*N*)	Cluster 2 (*N*)	*P-*value
Did you perform animal experiments during studies directly?	Yes	86	50	0.83^c^	56	47	0.55^c^
	No	9	5		1	1	
Do you kill animals personally	Yes	60	41	0.21^a^	42	28	0.13^a^
	No	33	14		15	19	
Do you see the possibility of resignation from animal experiments	Yes	13	2	**0.045**^b^	54	38	**0.028**^b^
	No	80	53		3	9	
Are animal experiments inevitable?	Yes	81	50	0.71^b^	54	39	**0.049**^c^
	No	10	5		2	8	
Should animal experiments be currently carried out?	Yes	9	3	0.51^c^	0	7	**0.003**^d^
	No	79	51		54	38	
Is animal experimentation unnecessary?	Yes	45	21	0.32^a^	18	28	**0.01**^a^
	No	47	31		36	18	
Experiments on animals cause an emotional burden	Yes	71	33	**0.042**^a^	32	41	**0.0004**^a^
	No	20	20		24	5	
Do you have remorse over the killing of animals?	Yes	54	20	**0.013**^a^	17	31	**0.0002**^a^
	No	36	32		38	14	
Did you ever avoid animal experiments?	Yes	41	11	**0.003**^a^	10	26	**<0.0001**^a^
	No	48	41		44	18	
**Accompanying feelings (procedures performed on animals during studies)**							
Curiosity	Yes	49	34	0.24^b^	50	33	**0.017**^a^
	No	16	6		7	15	
Fascination	Yes	21	16	0.42^a^	25	12	**0.044**^a^
	No	44	24		32	36	
Reluctance	Yes	9	3	0.50^c^	0	12	**<0.0001**^d^
	No	56	37		57	36	
Compassion for animals	Yes	34	10	**0.007**^a^	2	41	**<0.0001**^a^
	No	32	30		55	7	
Postponing	Yes	2	0	0.38^d^	0	2	0.21^d^
	No	63	40		57	46	
Change with another researcher	Yes	7	2	0.51^c^	0	9	**0.0006**^d^
	No	58	38		57	39	
Apprehension	Yes	9	5	0.92^c^	1	13	**0.0002**^b^
	No	56	35		56	35	
Anxiety	Yes	11	2	0.13^c^	2	11	**0.003**^b^
	No	54	38		55	37	
Other	Yes	8	7	0.46^b^	11	4	0.11^b^
	No	57	33		46	44	
**Accompanying feelings (procedures performed on animals after graduation)**							
Relief	Yes	18	14	0.25^a^	11	11	0.66^a^
	No	72	35		42	34	
Satisfaction with procedures	Yes	67	30	0.1^a^	37	32	0.89^a^
	No	23	19		16	13	
To feel sorry for animals	Yes	22	20	**0.036**^a^	19	22	0.37^a^
	No	68	28		34	32	
Remorse	Yes	5	8	0.08^c^	7	2	0.25^c^
	No	85	41		46	43	
Annoyance	Yes	1	4	**0.034**^c^	2	1	0.89^c^
	No	89	45		51	44	
Helplessness	Yes	2	2	0.92^c^	2	1	0.89^c^
	No	88	47		51	44	
Indifference	Yes	1	1	0.76^c^	1	1	0.55^c^
	No	89	48		52	44	
Curiosity of research results	Yes	62	32	0.67^a^	34	32	0.46^a^
	No	28	17		19	13	
Other	Yes	6	3	0.81^c^	4	4	0.90^c^
	No	84	46		49	41	

**Notes:**

a Pearson χ^2^ test.

b V^2^ test.

c Yates corrected χ^2^ test.

d Fisher exact test.

N, number of respondents. *P* values < 0.05 were indicated in bold.

Experiments on animals cause a stronger emotional burden on women compared to male respondents. A total of 78% of women said that animal testing causes an emotional burden. Among men, the percentage was 62%. 22% of women and 34% of men, respectively, do not associate emotional burden with animal experiences (*P* = 0.042).

It was also found that there is a relationship between animal experiments and sex-dependent remorse felt by respondents, and more women declared remorse (60%). Among men, this percentage was 38%, while the remaining 62% of men did not declare remorse (*P* = 0.013).

A significant larger percentage of men do not avoid animal testing (78%), the answers among women were almost equal, and 46% of females stated that they most often avoid animal testing (*P* = 0.003).

When analyzing compassion for animals, in the group of women, the responses were more or less equal (52% declared compassion, 48% did not). In the group of men, 25% indicated the affirmative answer, while 75% indicated the answer “no” (*P* = 0.007).

Answers regarding feelings sorry for animals, 76% of women surveyed did not indicate that they felt sorry for animals. In the case of males, 42% feel regret, 58% said they do not feel sorry to animals (*P* = 0.036).

The respondents did not show an increased level of annoyance in connection with conducting animal experiments. In this case, as much as 99% of women and 91% of men were not annoyed by conducting animal experiments. However, considering the other respondents, men declaring irritation appeared more often (8%) than women (1%) (*P* = 0.034).

## Discussion

### Animals use in the experiments

In 2017, over 9 million animals were used for scientific and other experimental purposes in the European Union. Around 75% were rodents and rabbits; 61% of the total number of animals were mice (The European Parliament and the Council of the European Union, 2020). In presented study 63% were rodents and medium-sized (including rabbits) animals, and murine accounted 56%. The total number of animals used in research in 2018 in Germany was 2.1 million, a similar level to 2015 and 2016. Germany ranks second after the UK in terms of animal use with a total of 3.3 million animals used in 2017 (The European Association for Animal Research (EARA), 2019). In 2018, 3.52 million procedures were carried out in Great Britain involving living animals. This is a decrease of 7% on last year, and the lowest number of procedures since 2007. According to the Report of the Supreme Audit Office in Poland, less than 150,000 vertebrates, mainly mice and rats, die annually in scientific research. Over 41,000 animals were used in experiments of the highest severity category determined in accordance with 2010 EU directive (Supreme Audit Office of Poland—Department of Science Education and National Heritage, 2017). In 2015, 28,921 animals were subjected to severe experiments (National Ethics Committee for Animal Experiments, 2015). Between 2011 and 2014, 18,542 animals were subjected to such procedures and in 2014, six animals were subjected to a procedure causing extreme suffering. In 2017, 51,279 animals were used for studies of the highest severity category (National Ethics Committee for Animal Experiments, 2017). Therefore, animal experimentation is still common. The performance of such research is not indifferent to the psyche of the scientists involved. Although our research has shown that between 83% and 96% of respondents (depending on their approach to animal experimentation during their studies) believe that animal testing is inevitable, it often causes negative emotions in researchers. A total of 72% of those surveyed clearly stated that performing experiments on animals is emotionally burdening for them. A total of 63% of those surveyed felt the stress of performing animal procedures. However, 47% of those surveyed admitted that many experiments on animals are unnecessary. A survey conducted in Portugal indicates that the assessment of the need for animal experimentation is not related to the knowledge on ethical aspects of experimentation (Franco & Olsson, 2014). The assumption that research is necessary and the lack of ethical reflection may be related to the alleviation of psychological tension accompanying the performance of procedures on animals. In our study this assumption is unfounded for nearly half of the participants and our research shows that experimenters feel the stress of carrying out procedures on animals (Fig. 4). It has been shown that women are much more stressed by animal experimentation than men. Similar conclusions were reached by researchers from Ewha Womans University (Kang et al., 2018). Such an outcome can be explained both by biological aspects (De Visser et al., 2010) and by socio-cultural expectations of a higher level of empathy and sensitivity in women than in men. In our research, the answers of the respondents allowed us to assign them to two clusters in which they differed in the intensity of the procedures performed on animals. Differences in the level of stress involved in killing animals have been shown. The respondents in the first cluster, who were performing less invasive experiments, showed a lower level of stress in relation to the second cluster, where the respondents felt a much higher level of stress (Fig. 5). The psychological tension that accompanies the execution on animals of more or less but always painful procedures in scientific experiments can have a far-reaching negative impact. As our research indicated, for many of the respondents, animal experimentation is associated with emotionally difficult experiences. Such tensions always have to be accompanied by some kind of psychological defensive mechanism to reduce the stress. However, long-term stress, as Selye (1976) pointed out, leads to the depletion of the body’s resources, which may affect the broadly understood (not only professional) functioning of people experiencing on animals. It should be stressed that the effects of the psychological burden of negative emotions resulting from experimenting on animals, according to Selye’s concept (but also other concepts of stress), take the form of psychological processes but also somatic phenomena. They are closely related to each other, although this relationship is not always clear to people assessing the condition of people suffering from chronic stress.

If an individual perceives animal experimentation as stressful, it should be regarded as a long-term stressor’s impact. The situation of experiencing is a repeated sequence of actions in which people inflict suffering on animals, then observe this suffering and finally lead to the death of the animal. A sensitive experimenter has to activate some psychological mechanisms to cope with such a situation and his creative effectiveness at work may be reduced by experiencing uncomfortable emotional tension.

Negative psychological aspects of animal experimentation may involve several dimensions. In the first place, it is the individual dimension - concerning the experimenter himself, his psychological balance and his sense of individual well-being. In the second place, it concerns the immediate social environment which are the people closest to the experimenters. The third dimension is the professional scientific community. The fourth dimension is the broadly understood social environment and the public opinion. Emotional and psychological difficulties that accompany scientists may affect the creation of the image of experimental sciences as such, whose undertaking requires problematic behaviour towards animals.

### Sources of emotional tension

The emotional tension experienced by people experimenting on animals can have several sources. First of all, it results from the empathy of the experimenter, who experiences a dissonance between the reading of pain signs in the animal and the need to continue the experiment. Paradoxically, animal experimentation requires constant observation of changes in animals’ behaviour and appearance. Obviously, this requires sensitivity to nuances appearing in behaviour and in the psychosomatic dimension. On the other hand, this sensitivity is accompanied by the necessity to continue experiments and therefore cause suffering to animals. This is a very serious stressor.

A second source of experiencing the stress is the growing pressure from the public opinion and the social environment, which increasingly clearly and loudly questions the need for animal experimentation, monitors these experiments and doubts their ethics and validity. Activism in this area seems to be increasing and it certainly affects the consciousness of people who experiment on animals.

A third stressor is the peer pressure of the scientific community, which anticipates animal experimentation. In addition to real, realistic and straightforwardly formulated expectations, the experimenters may also be under pressure from their own perceptions of what other people expect. As indicated by numerous phenomena described in psychology and especially in social psychology, the specificity of human behaviour is adjusted not so much to the real expectations of the environment, but to the ideas that people have about these expectations. A total of 6% of the respondents participating in our study pointed out that they feel pressure from the scientific community to experiment on animals, and assessed this pressure as burdensome.

Another element of animal experimentation that generates stress is the awareness of the real financial costs involved with conducting experiments and the feeling that these experiments should bring measurable benefits. This thesis is clearly confirmed by our research, as 68% of the respondents said they felt fearful about the outcome of the experiment (Table 2). Unfortunately, we do not have data regarding other reasons, such as if they were afraid of losing their livelihood source.

The sources of stress indicated above should be viewed in an additional context, namely in the context of contradictions between the stressors. Staying in a situation of conflicts where contradictory expectations and experiences collide is a hardship in itself. As we pointed out above, psychologically speaking, animal experimenters are in stalemate situations (empathic observation of suffering versus inflicting further suffering; willingness to conduct scientific experiments versus resistance and the public opinion’s pressure; reluctance to conduct animal experiments versus the necessity to continue a career).

### Coping with stress

Coping with stress can take the form of three different styles, depending on your personality profile and your situation. There are three basic coping styles: task-oriented, emotion-oriented, and avoidance-oriented (Parker & Endler, 1990). In turn, according to Carver & Scheier (1994), stress management strategies can be divided into constructive strategies (active coping, i.e. taking actions rationally; planning, i.e. focusing on the programming of the process itself and not on its stress-causing aspects; suppressing of competing activities, i.e. eliminating threads and elements that potentially disrupt the undertaken activities; self-control – refraining from impulsive action; positive reinterpretation and development; seeking instrumental or emotional social support, distracting attention from the stressful phenomenon by turning to religion; acceptance of the phenomenon; focus on and venting of emotions; but also more risky: denial, mental or behavioural disengagement or distancing oneself from the problem of *"disengagement"*; using drugs or alcohol).

The respondents surveyed by us pointed to the use of some of these techniques. Avoidance—i.e., for example, exchanging the execution of the experiment/exercise with another person (9% of the respondents), postponing the execution of the experiment (2% of the respondents) can be considered as behavioral disengagement from the burden (Fig. 1). One-third of those surveyed indicated that they avoided some animal experimentation (Table 2).

Taking into consideration the correlation matrix analysis and feelings that appeared in respondents during studies and after completed research protocol, two kinds of researches could be distinguished. On the one hand, during the studies, the compassion for animals and apprehension was dominant, and on the other hand, curiosity was intensified. After the research protocol, the novelty and satisfaction from the conducted procedures strikeout in the foreground, but at the opposite extreme were annoyance and indifference (Fig. 1). Those observations were supported by tree clustering, which reveals two clusters differed in the accompanying feelings during studies. Curiosity and fascination covered other perceived feelings, which can be described as more positive, animal welfare-oriented. In this case, we could think about two different psychological types of people, self-oriented and other-oriented. Both groups could be distinguished by the diversity of approach to animal experiments. But this could be explained by the “curiosity of exploration” which it seemed like a significant driving force of progress and could fill the knowledge gap. Different emotional burden leads to diverse coping with problems and emotional approach. Thus, the greater the personal compassion for animals, the curiosity and fascination fade into the background.

In the context of the welfare of animals used in experiments, the most dangerous techniques are those that take different forms of psychological distancing from the phenomenon. This is because it can lead (and probably in some cases leads) to the instrumental exploitation of animals, without taking into account their welfare and quality of life. On the other hand, the most stressful issue for the workers is the disengagement of suicide. Handling (The European Parliament and the Council of the European Union, 2010) should be used in all experiments, regardless of their length and severity. As our research has shown, the conscious use of handling is related to the increase in stress levels in the experimenters (Fig. 3B). The necessity to domesticate the animal (relating to mutual relationship between animals and humans) may cause the experimenter to see a living creature in the animal and not only the object of research. The research carried out by Franco, Sandøe & Olsson (2018) clearly indicates the necessity to train experimenters in the handling procedures. Procedures connected with handling and animal welfare should also be included in training programmes for experimenters (Nevalainen et al., 2000). Additionally, we found an average, inversely proportional, statistically significant correlation between the invasiveness of procedures and the perception of stress (Fig. 3): As the intensity of procedures decreased, the level of stress of respondents increased. Respondents experience more stress during mild than severe experiments. This may be due, inter alia, to the fact that in severe procedures, experimenters have the possibility (referring to the 3R—Replacement, Reduction and Refinement principles, introduced in 1959) (Russell & Burch, 1959; Mepham, 2008) to introduce improvements that alleviate the severity of procedures for animals. Thus, training in laboratory animal science is a valuable means of educating and raising awareness about animal welfare. These principles were introduced both to improve animal welfare and to improve the quality of experiments (Franco & Olsson, 2014). The mild experiments are the least invasive of the procedures, which makes additional improvements difficult. In the case of acute experiences, experimenters are more able to replace the tests they have used so far (e.g., they may abandon the Porsolt test in the case of research on depression) with more contemporary ones, which are less ethically controversial. The application of the 3Rs, as opposed to handling, entails changes to the experiment protocol and does not require additional contact with the animal. According to the results of the research presented by Franco *et al*., the vast majority of experimenters see the need to apply the 3Rs principles and implement them in their experiments, but the majority did not want additional training in this area. The research carried out in Portugal, Germany, Switzerland and Denmark (Franco & Olsson, 2014) confirms our thesis that the application and knowledge of the 3Rs principles not only (potentially) improves animal welfare but above all the welfare of the experimenters. The reluctance of the experimenters to explore knowledge about improvements in experiments can be seen as a confirmation of the fact that they consider the welfare of the experimenters to be more important than that of the animals. This also confirms our thesis about the negative psychological effects of conducting experiments.

As previously indicated on the example of representatives of veterinary sciences, the stress in professions related to animal procedures is an important factor affecting the quality of life of people undertaking this type of work. The lack of such support may result in a decline in creativity and creative initiative among researchers, professional burnout, and even suicide (Tomasi et al., 2019).

## Conclusions

The research showed that there is a statistically significant group of respondents among those conducting animal experiments, who experience a significant level of stress resulting from the obligation to conduct experiments on sentient and living organisms. Scientists conducting such experiments feel remorse, emotional tension and a sense of helplessness. During the research, this group of respondents avoided performing animal procedures, usually exchanging with another person. Many respondents believe that animal experimentation is not necessary in their scientific research, but they feel pressured to perform it. The discomfort experienced by scientists due to the need to experiment on animals can have a negative impact on their well-being, creativity and commitment to work. It can also create a negative image of science in the broader public opinion. It therefore appears that reducing animal experimentation is important from the point of view of animal and human welfare.

## Supplemental Information

10.7717/peerj.11035/supp-1 Supplemental Information 1 Survey (Polish).

10.7717/peerj.11035/supp-2 Supplemental Information 2 Survey (English).

10.7717/peerj.11035/supp-3 Supplemental Information 3 Raw data.

## Additional Information and Declarations

### Data Availability

The following information was supplied regarding data availability: Raw data are available as a Supplemental File.
