# Supplementary material for: Negative psychological aspects of working with experimental animals in scientific research
Source: PeerJ. 2021 Apr 20;9:e11035. doi: 10.7717/peerj.11035 (PMC8063873; doi:10.7717/peerj.11035)
Supplement: Supplemental Information 1 [file peerj-09-11035-s001.pdf]

Wyrażam zgodę na udział w badaniu. W pełni rozumiem koncepcję tych badań. Zostałem poinformowany/a, że wszystkie informacje pobrane z badań zostaną zakodowane w celu ochrony danych każdego z uczestników badania. Żadne nazwiska ani inne informacje identyfikujące nie będą wykorzystywane podczas omawiania lub zgłaszania danych. Rozumiem również, że moje odpowiedzi mogą być zgłaszane w pracy i prezentacji, a moja tożsamość będzie zachowana w tajemnicy i nie będą umieszczane żadne informacje identyfikujące mnie.

Za mój udział nie przysługuje mi żadne wynagrodzenie. Rozumiem, że mój udział w tym projekcie jest całkowicie dobrowolny. Mogę się wycofać w dowolnym momencie przed lub w trakcie procedury badawczej.

Czy podczas studiów wykonywał/a Pan/i bezpośrednio jakiekolwiek procedury na żywych zwierzętach?
Tak
Nie

Jeśli tak- jakie to były procedury?
………

Jeśli wykonywał/a Pan/i procedury na zwierzętach w czasie studiów, jakie odczucia towarzyszyły Panu/i podczas ich trwania? Proszę zakreślić wszystkie adekwatne odpowiedzi
Fascynacja
Zaciekawienie
Niechęć do wykonania ćwiczenia
Współczucie dla zwierząt
Dążenie odwleczenia ćwiczenia w czasie
Chęć zamiany z inną osobą, która wykona ćwiczenie za mnie
Obawa
Lęk
Inne- jakie? ………………………………………………………………………………………

Czy w czasie studiów zdarzało się Panu/i unikać procedur na zwierzętach? Jeśli tak- to jak?
Planowana nieobecność na zajęciach
Zamiana z inna osobą
Odmowa wykonania ćwiczenia
Wykonanie innego zadania „w zamian"
Inne- jakie? ………………………………………………………………………………………

W którym roku przeprowadził/a Pan/i pierwsze doświadczenie z udziałem zwierząt?
………

Proszę o opisanie (bardzo ogólnie) jaki to był rodzaj doświadczenia?
………

Jakie zwierzę/ta były częścią procedury badawczej?
………

Ile zwierząt uczestniczyło w badaniu?
………

Jakie odczucia pojawiły się u Pana/i po zakończonym protokole czynności badawczych?

Ulga
Zadowolenie z dobrze wykonanych procedur
Żal zwierząt
Wyrzuty sumienia
Irytacja
Bezradność
Obojętność
Ciekawość wyników
Inne- jakie? ……………………………………………………………………………………

Czy osobiście uśmierca Pan/i zwierzęta?
Tak
Nie

Uśmiercanie zwierząt wiąże się z pojawieniem się u Pana/i stresu
Zdecydowanie tak
Raczej tak
Trudno powiedzieć
Raczej nie
Zdecydowanie nie
Inne- jakie? ……………………………………………………………………………………

Jeśli tak - czy może Pan/i wskazać dlaczego?
Obawa przed tym czy doświadczenie się uda
Świadomość odpowiedzialności za wydatkowane środki
Obawa przed oceną ze strony współpracowników
Poczucie odpowiedzialności za życie zwierząt
Obawa przed agresywną reakcją zwierzęcia
Obawa przed uszkodzeniem własnego ciała (np. podczas dekapitacji gilotyną
Inne- jakie? ……………………………………………………………………………………

Dostrzegam pozytywne znaczenie oswajania/handlingu w eksperymentach na zwierzętach?
Zdecydowanie tak
Raczej tak
Trudno powiedzieć
Raczej nie
Zdecydowanie nie
Inne – jakie? ……………………………………………………………………..

Jak proces handlingu wpływa na Pana/i na relację ze zwierzętami?
Dobrze wykonany handling uspokaja mnie
Czuję się z nim komfortowo
Dzięki handlingowi zżywam się ze zwierzętami
Przez handling trudniej mi wykonywać procedury
Nie ma wpływu na relację ze zwierzętami
Irytuje mnie marnowanie czasu
Handling nie robi na mnie żadnego wrażenia
Inne – jakie? ……………………………………………………………………..

Jakie odczucia towarzyszyły Panu/i w związku z wprowadzeniem obowiązkowych urozmaiceń do środowiska zwierząt eksperymentalnych?
Zdecydowanie pozytywne- to potrzebna innowacja

Raczej pozytywne
Trudno powiedzieć
Raczej negatywne
Zdecydowanie negatywne- to strata czasu / pieniędzy
Inne – jakie? ……………………………………………………………………..

Czy planuje Pan/i realizowanie dalszych procedur z udziałem zwierząt laboratoryjnych, (pytanie wielokrotnego wyboru)
Zamierzam kontynuować doświadczenia z udziałem modelu zwierzęcego
Zamierzam stosować jedynie kultury In vitro
Nie zamierzam kontynuować badań z udziałem modeli zwierzęcych
trudno powiedzieć
Inne- jakie? ……………………………………………………………………………………

Proszę wskazać czy poniższe zdania ocenia Pan/i jako prawdziwe (P/F)
Nie ma współcześnie możliwości odejścia od doświadczeń naukowych na zwierzętach.
Doświadczenia na zwierzętach są nieuniknione.
Doświadczenia na zwierzętach nie powinny być już dzisiaj przeprowadzane.
Uważam, że duża część doświadczeń na zwierzętach jest zbędna.
Doświadczenia na zwierzętach powodują obciążenie emocjonalne.
Mam wyrzuty sumienia z powodu uśmiercania zwierząt.
Wykonuję doświadczenia na zwierzętach, bo jest na to presja środowiska.
Najchętniej unikałabym/unikałbym prowadzenia doświadczeń na zwierzętach.
Moje badania na zwierzętach są wartościowe dla międzynarodowego środowiska naukowego
Inne komentarze? ……………………………………………………………………………………

Jaką dyscyplinę/dziedzinę nauki Pan/i reprezentuje?
………

Pana/i płeć:
Kobieta
Mężczyzna

Wiek
Do 30
31-40
41-50
51-60
60 i więcej

Ukończone studia
………
