# Supplementary material for: Negative psychological aspects of working with experimental animals in scientific research
Source: PeerJ. 2021 Apr 20;9:e11035. doi: 10.7717/peerj.11035 (PMC8063873; doi:10.7717/peerj.11035)
Supplement: Supplemental Information 2 [file peerj-09-11035-s002.pdf]

I agree to serve as a subject in the research mentioned above. I understand the concept of this research. All information taken from the study will be coded to protect each subject's name. No names or other identifying information will be used when discussing or reporting data. I also understand that my responses may be reported in the paper and presentation, and my identity will be kept confidential, and no identifying information about me will be included. I understand that the risks to me are minimal.

There will be no compensation of any kind available for my participation. I understand that my participation in this project is completely voluntary and that my choice of whether to participate in this project. I am free to withdraw at any point before or during the research procedure.

Did you perform any procedures on live animals directly during your studies?
Yes
No

If so, what were the procedures?
…….

If you have conducted scientific procedures on animals during your studies period, what feelings have you experienced? Please indicate all the true answers.

Fascination
Curiosity
Reluctance to do the exercise
Compassion for animals
Aiming to postpone the exercise in time
Wanting to swap with another person who will do the exercise for me
Fear
Anxiety
Others- what kind? …………………………………………………………………………………

Have you tried to avoid conducting procedures on animals during your studies period? If yes- how did you do it?

Planned absence from classes
Switch with another person
Refusal to perform the exercise
Performing another task "in return"
Others- what kind? …………………………………………………………………………………

Which year did you conduct your first experiment using animals? Please describe the brief type of experiment.
…….

Which animals were used in experimental procedures? How many animals were used?

…….

What feelings have you experienced after finishing the research protocol on animals?

Relief
Satisfaction with well-executed procedures
Animal regret
Remorse
Irritation
Helplessness
Indifference
Curiosity about the results
Others- what kind? ………………………………………………………………………………

Do you personally sacrifice animals?

Yes
No

Killing animals is stressful for me
Definitely yes
Yes
Hard to say
Rather no
Definitely no
Other……

If yes- can you indicate why
Fear of the experience succeeding
Awareness of responsibility for the funds spent
Fear of evaluation by colleagues
Feeling responsible for the lives of animals
Fear of aggressive reaction of the animal
Fear of damaging your own body (e.g., during decapitation with the guillotine)
Others- what kind? ………….

I see handling's positive impact on experiments with animals.

Definitely yes
Rather yes
Hard to say
Probably no
Definitely no
Others- what kind? …………………………………………………………………….

How handling effects your relations with animals?
Well done handling calms me down
I feel comfortable doing the handling
Thanks to the handling, I'm able to handle the animals
The handling makes it more difficult for me to perform procedures
Does not affect the relationship with animals

I'm annoyed by the waste of time
Handling does not impress me at all
Others - which ones?

What feelings have you experienced when regulations imposed obligatory use of environmental enrichment for experimental animals?

Definitely positive - a necessary innovation
Rather positive.
It's hard to say
Rather negative.
Definitely negative - a waste of time / money
Others - which ones?

Do you plan to carry out further procedures involving laboratory animals? (multiple choice question)
I intend to continue the experiments with the animal model
I intend to use only in vitro cultures
I don't intend to continue research with animal models
It's hard to say
Others- what kind? ……………………………………………………………………………………

Please indicate whether the following sentences are true. (Mark T/F)
There is no way to move away from animal experiments nowadays.
Animal experiments are inevitable.
Animal experiments should no longer be conducted today.
I believe that a large part of animal experimentation is superfluous.
Animal experiments cause emotional strain.
I feel guilty about killing animals.
I perform experiments on animals because there is pressure from the environment.
I would most willingly avoid animal experiments.
My research on animals is valuable to the international scientific community.
Other comments? ……………………………………………………………………………………

What scientific discipline do you represent?

……….

Gender
Women
Men

Age
< 30
31-40
41-50
51-60
60 <

Direction of studies
